# Amino Acid Profile Alterations in Phenylketonuria: Implications for Clinical Practice

**DOI:** 10.3390/metabo14070397

**Published:** 2024-07-21

**Authors:** Eliza Matuszewska, Joanna Matysiak, Łukasz Kałużny, Dariusz Walkowiak, Szymon Plewa, Monika Duś-Żuchowska, Natalia Rzetecka, Małgorzata Jamka, Agnieszka Klupczyńska-Gabryszak, Marcin Piorunek, Jan Matysiak, Jarosław Walkowiak

**Affiliations:** 1 Department of Inorganic and Analytical Chemistry, Poznan University of Medical Sciences, 3 Rokietnicka Street, 60-806 Poznan, Poland; eliza.matuszewska@ump.edu.pl (E.M.); splewa@ump.edu.pl (S.P.); natalia.rzetecka@student.ump.edu.pl (N.R.); aklupczynska@ump.edu.pl (A.K.-G.); 2 Faculty of Health Sciences, Calisia University–Kalisz, 62-800 Kalisz, Poland; j.matysiak@uniwersytetkaliski.edu.pl; 3 Department of Pediatric Gastroenterology and Metabolic Diseases, Poznan University of Medical Sciences, Szpitalna Str. 27/33, 60-572 Poznań, Poland; lukasz@jerozolima.poznan.pl (Ł.K.); mduszuchowska@ump.edu.pl (M.D.-Ż.); mjamka@ump.edu.pl (M.J.); piorun.mp@gmail.com (M.P.); 4 Department of Organization and Management in Health Care, Poznan University of Medical Sciences, Przybyszewskiego Str. 39, 60-356 Poznań, Poland; dariuszwalkowiak@ump.edu.pl

**Keywords:** liquid chromatography–tandem mass spectrometry, kynurenine, tyrosine, asymmetric dimethylarginine, creatinine

## Abstract

Patients with phenylketonuria (PKU) must restrict their intake of phenylalanine, which can also affect the levels of other essential and non-essential amino acids due to inadequate supply. Therefore, our objective was to assess amino acids in serum samples from 20 PKU patients and compare them with results from 51 healthy subjects. A sample analysis was conducted using liquid chromatography–tandem mass spectrometry. We obtained levels of 28 substances, including amino acids, biogenic amines, carnitine, and acetylcarnitine. Kynurenine (*p* = 0.000001), tyrosine (*p* = 0.0002), asparagine (*p* = 0.001), proline (*p* = 0.012), and the kynurenine/tryptophan ratio (*p* < 0.000001) were identified as features that differed between the studied groups, being significantly lower in patients with PKU. Glycine (*p* = 0.000012), putrescine (*p* = 0.0055), asymmetric dimethylarginine (*p* = 0.01), creatinine (*p* = 0.035) levels, as well as the total level of glucogenic amino acids (*p* = 0.0018), and the ratios of putrescine/ornithine (*p* = 0.003) and citrulline/ornithine (*p* = 0.0043) were significantly higher in the PKU group. In conclusion, the amino acid profiles in patients with PKU differ significantly from those in healthy peers, with potential clinical implications. These findings confirm the importance of metabolic testing in clinical practice and highlight the necessity for adequate dietary monitoring and adjustment.

## 1. Introduction

Phenylketonuria (PKU, OMIM 261,600) is a hereditary metabolic disorder caused by a single gene mutation and is inherited in an autosomal recessive manner [1]. The disease stems from a deficiency or insufficiency of the enzyme phenylalanine hydroxylase (PAH, EC 1.14.16.1), which is responsible for converting phenylalanine (Phe) into tyrosine. Most PKU cases result from mutations in the *PAH* gene, leading to the misfolding and instability of the PAH enzyme [2]. This enzyme dysfunction causes an elevation in Phe levels in the blood and a shortage of tyrosine, leading to the toxic buildup of Phe in the brain. This metabolic process occurs within liver cells, where Phe serves as a substrate and is converted by PAH, molecular oxygen, iron, and a non-protein cofactor called tetrahydrobiopterin (BH4). The *PAH* gene, responsible for encoding the PAH enzyme, is located on chromosome 12 (q22–q24) [3]. To date, 3370 *PAH* variants have been identified, each affecting the enzyme’s activity in different ways [1,4].

PKU has a global impact, with an estimated 0.45 million individuals affected, and its prevalence is roughly 1 in 23,930 live births. However, the rates of occurrence vary among different countries [5]. In Poland, PKU occurs at a frequency of 1 in 8309 individuals [6]. The observed variation in the phenotypic presentation of PKU can be attributed to a combination of genetic factors, individual predispositions, such as the blood–brain barrier, and environmental influences. Among Europeans, PKU stands as the most prevalent inborn error of metabolism. Notably, PKU played a significant role in pioneering the development of effective treatments for disorders that cause both physical and cognitive impairments [7].

The primary approach to treating PKU involves implementing a Phe-restricted diet, which necessitates reducing the intake of natural proteins and substituting them with a protein source that lacks Phe, consisting of a combination of amino acids. Protein substitutes supply the fundamental components for the formation of tissue proteins, and their amino acids are crucial for the creation of hormones, enzymes, and various cellular functions [8]. This therapeutic regimen should be overseen by a knowledgeable physician who specializes in metabolic disorders, along with a team of dietitians [6,8]. Since the foods typically consumed for protein also contain essential nutrients, it is crucial for a Phe-restricted diet to include alternative sources that provide all the necessary nutrients for healthy growth and well-being. In certain cases, patients may also require tyrosine supplementation since it is typically scarce in the PKU diet, ensuring the sufficient production of neurotransmitters. However, the rigorous nature of the PKU diet can pose challenges for patients and their families in maintaining consistent compliance over the long term [9,10]. Furthermore, despite adhering strictly to the diet, some individuals with PKU may still encounter neurological symptoms, emphasizing the necessity for additional treatment options [11]. A study revealed significantly higher rates of autism spectrum disorders and eating disorders among adults with PKU compared to the general population [12]. Similarly, elevated rates of depression and anxiety were observed in the overall PKU population [13,14]. Another study demonstrated that adolescent and adult PKU patients exhibited poorer social cognition and social skills compared to control groups [11]. There was also a tendency towards lower or delayed autonomy [15] and emotional difficulties associated with adhering to a PKU diet [16]. Furthermore, PKU patients often experience feelings of social isolation and limitations in social interaction [17]. Many of them also face significant neurocognitive, mental health, and general health challenges [13,15,18]. Additionally, there are reports that overweight and obesity may be more common among PKU patients when compared to their healthy peers [19,20]. Research is currently underway to develop alternative therapies for PKU, including enzyme replacement therapies and gene therapies aimed at correcting the underlying genetic mutation. Additionally, researchers are exploring the use of pharmacological agents to enhance the activity of PAH and reduce the accumulation of Phe in the brain [21,22,23].

Upon an accurate diagnosis of the disease, patients are prescribed a low Phe diet. The regular monitoring of blood Phe levels is necessary to evaluate the efficacy of the elimination diet. However, the differences between PKU patients and healthy individuals extend beyond Phe concentrations in the body. The levels of other essential and non-essential amino acids may also be affected due to inadequate supply. Thus, the objective of this study was to quantify the concentrations of 28 amino acids in serum samples from PKU patients and compare them with results obtained from the control group. A sophisticated analytical system based on high-performance liquid chromatography–mass spectrometry was employed to achieve this. The findings from this study have the potential to enhance our understanding of the underlying mechanisms involved in PKU development. Furthermore, they may serve as a foundation for designing an even more tailored diet to address the intricate needs of PKU patients.

## 2. Materials and Methods

There was a cross-sectional study [24] comprising 20 patients with classical PKU treated at the Department of Pediatric Gastroenterology and Metabolic Diseases, Poznan University of Medical Sciences. The control group consisted of 51 healthy subjects (Table 1). For the purposes of the analysis, classical PKU was defined as a disease that, at diagnosis, required a low Phe diet to maintain plasma Phe levels within the target range of 2–6 mg/dL (120–360 μmol/L), and that, without diet, would result in Phe levels exceeding 20 mg/dL (1200 μmol/L) [25]. The other inclusion criteria for patients were diagnosis in the screening program and continuous treatment (Phe-restricted diet using Phe-free formula). The exclusion criteria, both for patients and healthy controls, included severe chronic or acute diseases, pregnancy, and breastfeeding. None of the patients with PKU received additional treatment (e.g., BH4 or PEG-PAL formulation).

All patients were recommended to perform monthly routine checks of Phe concentrations. All blood Phe concentrations collected during the 12 months preceding blood collection were evaluated in a dry blood spot; a venous blood sample (0.5 mL to clot) taken before eating in the morning following an overnight fast was checked using a fluorometric method. The Phe results were expressed as milligram per decilitre (mg/dL). The percentage of the Phe concentration within the therapeutic range was calculated for each patient according to recent recommendations [7].

The study design was compliant with the Helsinki Declaration [26] and was approved by the Bioethical Committee at the Poznan University of Medical Sciences (Poland) (approval number: 1119/18; 260/24). Written informed consent was obtained from all participants.

### 2.1. LC-MS-Based Determination of Amino Acid Serum Levels

A panel of metabolites was analyzed using a targeted metabolomics approach. The methodology was based on the AbsoluteIDQ p180 kit (Biocrates Life Sciences AG, Innsbruck, Austria), which enables the determination of up to 188 metabolites in each sample. The sample preparation comprised a few steps and was performed according to the manufacturer’s manual. The 96-well plate with pre-pipetted selected internal standards delivered by the manufacturer was used for sample preparation. During the first step, the phosphate buffer saline, quality control samples, calibrators, and serum were pipetted into the appropriate wells. Human samples were analyzed randomly using 10 µL of patients’ serum. Then, all spots were dried under the positive pressure nitrogen flow. After 30 min, 50 µL of derivatization agent was added, followed by 20 min of room-temperature incubation and another round of drying (60 min). Further, 300 µL of freshly prepared extraction solvent was pipetted to each well, and the plate was shaken (450 rpm) for 30 min. Then, the extract was passed through the filter layer to the capture plate and split into two separate 96-well plates. The content of each well was diluted with a suitable solvent adapted to the analysis of liquid chromatography–tandem mass spectrometry (LC-MS/MS) and flow injection analysis (FIA-MS/MS) and sealed with a silicone mat before placing it in a high-performance liquid chromatography (HPLC) autosampler.

The triple quadrupole tandem mass spectrometer 4000 QTRAP (Sciex, Framingham, MA, USA) coupled with HPLC 1260 Infinity (Agilent Technologies, Santa Clara, CA, USA) was used for metabolite determination. The quantification of amino acids and biogenic amines was preceded by chromatographic separation. The analyses of acylcarnitines were performed using flow injection analysis (FIA-MS/MS method). The instrument conditions were described by us earlier [27]. Data acquisition and quantification were performed under the control of Analyst 1.5.2 software (Sciex, Framingham, MA, USA) and MetIDQ software Version Boron (Biocrates Life Sciences AG, Innsbruck, Austria).

### 2.2. Minimum Sample Size

The minimum sample size was calculated based on tyrosine concentrations obtained in the study by Cannet et al. [28]. Calculations performed using the GPower 3.1.9.7 software (University of Kiel, Kiel, Germany) indicated that at least 34 participants are required to achieve a power of 80% (α = 0.05, β = 0.2). Assuming a 10% dropout rate, the minimal sample size is 19 individuals per group.

### 2.3. Data Analysis

To conduct statistical analyses, only features for which the measured concentration values were within the quantification range were subjected to statistical analyses. A value of *p* < 0.05 was considered statistically significant. Statistical analyses were conducted using Statistica 13.0 software (TIBCO Software Inc., Palo Alto, CA, USA) and MetaboAnalyst 5.0 (https://www.metaboanalyst.ca (accessed on 1 May 2024).

The variables were tested for normality based on the Shapiro–Wilk test. Data are expressed as medians and interquartile ranges and means ± standard deviations (SD) with 95% confidence intervals (95% CI). Based on the non-parametric data distribution, the Mann–Whitney U test was used to compare the PKU and control groups. The Fisher exact test was used to assess categorical variables [29]. 

A multivariate statistical analysis—the Partial-Least Squares Discriminant Analysis (PLS-DA)—was conducted to categorize the samples and generate a ranking of the investigated compounds that contribute to the classification of patients between two analyzed groups. The univariate receiver operating characteristic (ROC) curve and the area under the curve (AUC) were calculated to assess classification accuracy. In addition, Spearman coefficient correlations were calculated in the studied groups to evaluate the relationships between selected variables [30].

## 3. Results

The applied methodology resulted in the determination of serum levels of several amino acids and biogenic amines, as well as carnitine and acetylcarnitine. The concentrations of 28 analytes were measured within the quantification range.

The univariate statistics demonstrated that the statistically significant (*p* < 0.05) differences between the studied groups occurred in the levels of 12 measured features. In the PKU group, levels of kynurenine, tyrosine, asparagine, proline, and the kynurenine/tryptophan ratio were significantly decreased in comparison to the control group. On the contrary, glycine, putrescine, asymmetric dimethylarginine, and creatinine concentrations were significantly increased in the PKU group. Also, the total level of glucogenic amino acids and the ratios of citrulline/ornithine and putrescine/ornithine were significantly lower in the control group (Table 2).

**Figure 1:**
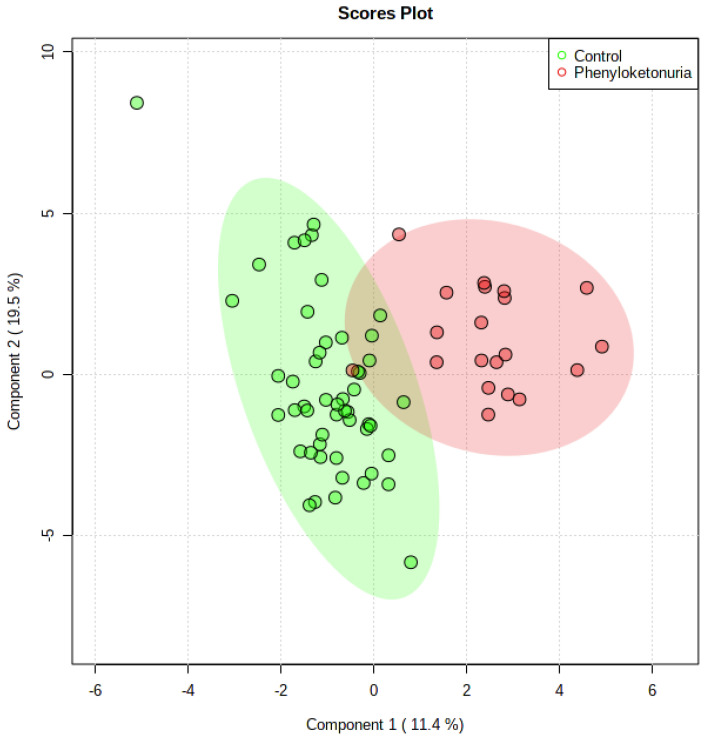
The Partial-Least Squares Discriminant Analysis (PLS-DA) score plot calculated for the measured features in two studied groups: phenylketonuria patients (red circles; *n* = 20) and controls (green circles; *n* = 51).

**Figure 2:**
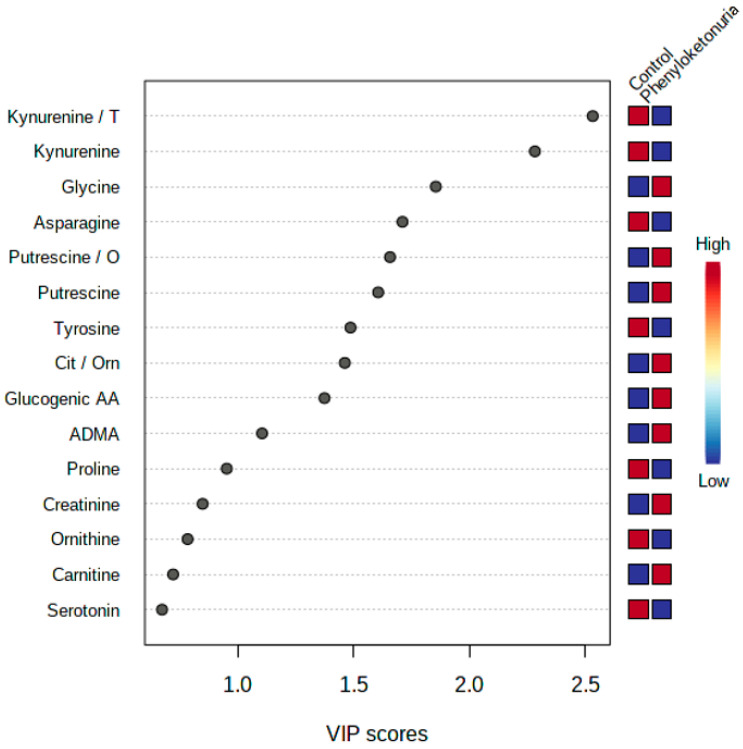
Important features identified using Partial-Least Squares Discriminant Analysis (PLS-DA). The boxes on the right indicate the relative concentrations of the corresponding features in each group under study. The diagram shows 15 features with the highest contribution in sample classification. ADMA—Asymmetric dimethylarginine; Cit—Citrulline; Glucogenic AA—Glucogenic amino acids; O/Orn—Ornithine; T—Tryptophan; and VIP—Variable Importance for Projection.

The results of the PLS-DA analysis indicate that the differences in concentrations of the measured features between the studied groups were strong enough to cause the grouping of the samples according to test or control group membership (Figure 1). The PLS-DA analysis depicts measured features with the highest ability to the grouping of the samples. As a result of the PLS-DA analysis, it has been confirmed that the variables selected in univariate statistics as discriminative between groups have, indeed, the best discriminative efficacy. Figure 2 shows the features listed according to their contribution to sample classification. The most differentiating variables were the kynurenine/tryptophan ratio, as well as kynurenine and glycine concentrations.

**Figure 3:**
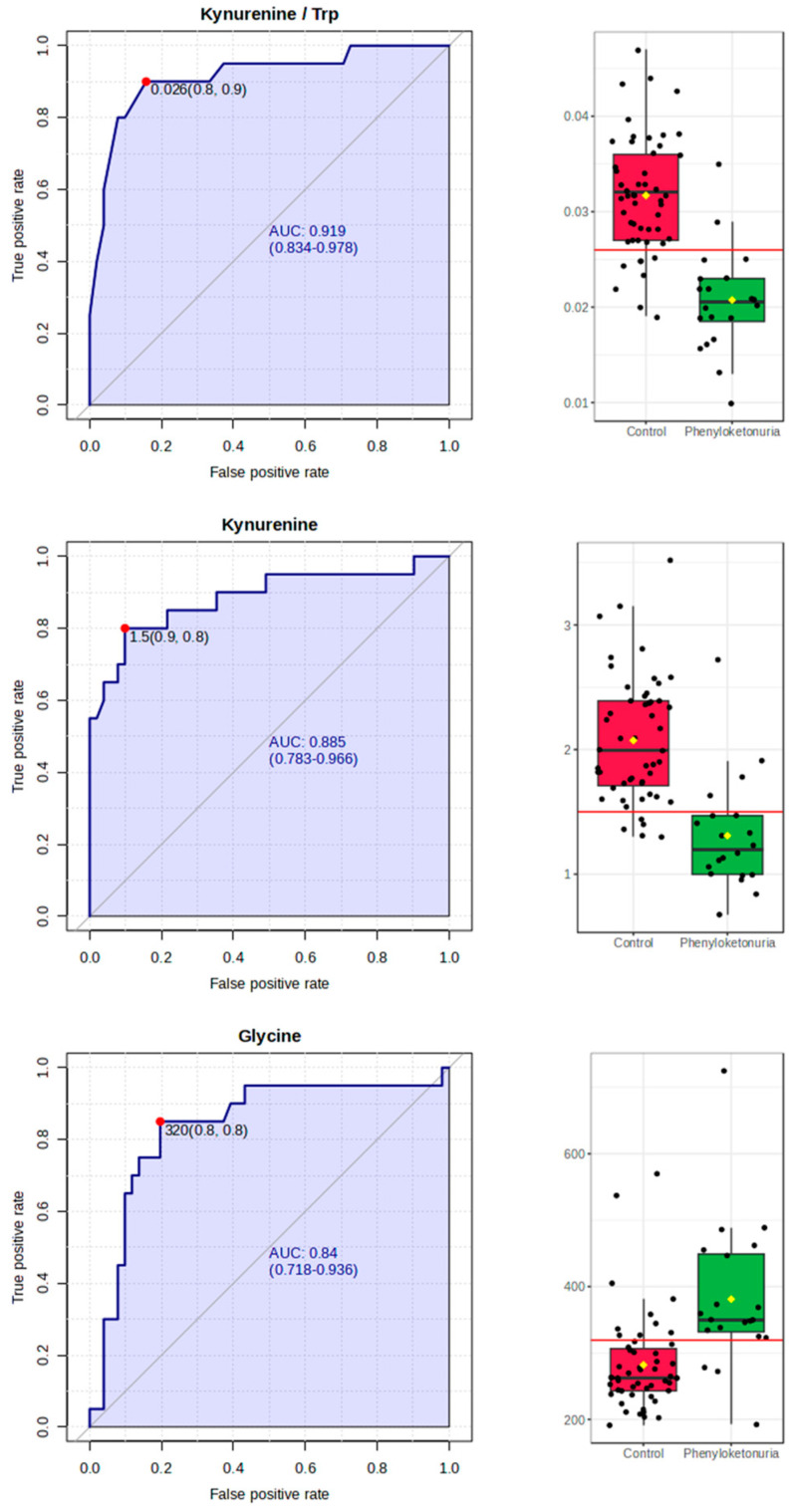
The receiver operating characteristic (ROC) curves with the highest area under the curve (AUC) values, and the box plots of the AUC with the optimal cut-off values calculated for the kynurenine/tryptophan ratio and for the concentrations of kynurenine and glycine.

The discriminative ability of the measured features that statistically significantly differentiate the studied groups was further checked by calculating the ROC curves. The ROC curve gives a graphical presentation of the sensitivity and specificity of the studied factors. According to the results of this study, the highest AUC value (0.919) was obtained by the kynurenine/tryptophan ratio, followed by kynurenine (0.885) and glycine (0.840) concentrations (Table 3 and Figure 3). These results are consistent with the data from the PLS-DA analysis. The lowest AUC value (0.663) was calculated for creatinine. AUC values for other measured characteristics, for which no statistically significant differences between groups were shown, were lower than the AUC calculated for creatinine.

**Figure 4:**
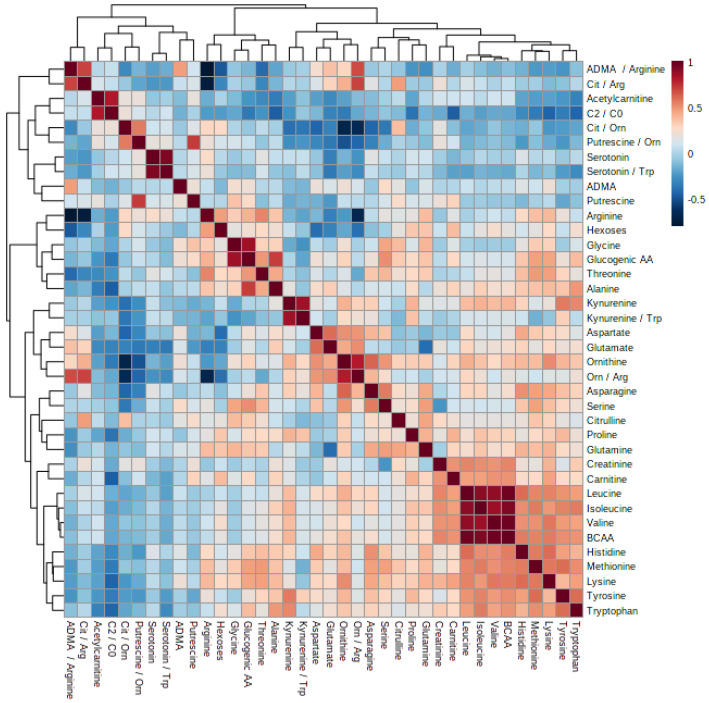
Heatmap and cluster analysis of the measured features in patients diagnosed with phenylketonuria and the control group. ADMA—asymmetric dimethylarginine; Arg—arginine; BCAA—branched-chain amino acids; Cit—citrulline; C2—acetylcarnitine; C0—carnitine; glucogenic AA—glucogenic amino acids; Orn—ornithine; and Trp—tryptophan.

The correlation heatmap was calculated using Rho Spearman’s algorithm to show the correlations between the measured features (Figure 4). The study revealed positive correlations between the levels of leucine, isoleucine, valine, and branched-chain amino acids. There was also a positive correlation between the concentration of glucogenic amino acids and the levels of glycine and alanine. Putrescine concentration was positively correlated with the putrescine/ornithine ratio. In addition, the ADMA/arginine and citrulline/arginine ratios showed positive correlations with the ornithine/arginine ratio, but negative correlations with the arginine concentration. The ornithine/arginine ratio was negatively correlated with both the arginine concentration and the citrulline/ornithine ratio.

## 4. Discussion

In this study, we analyzed 28 metabolic features in serum samples obtained from PKU patients and healthy controls. Our findings revealed significant reductions in the levels of four substances (asparagine, kynurenine, proline, and tyrosine) and the kynurenine/tryptophan ratio, and increased concentrations of four substances (asymmetric dimethylarginine, creatinine, glycine, and putrescine) as well as the total level of glucogenic amino acids and the ratios of citrulline/ornithine and putrescine/ornithine in the PKU group. Additionally, based on ROC curve analysis, the kynurenine/tryptophan ratio, kynurenine, glycine, and tyrosine levels showed the highest differential AUC value. These results underscore the importance of amino acid profile testing in clinical practice for patients with PKU.

PKU is one of the earliest described inborn errors of metabolism. Over the years, guidelines have been developed that clearly define the diagnostic process, which is now based on determining the Phe concentration and Phe/tyrosine ratio as part of newborn screening tests. While the amino acid analysis using the HPLC method remains useful in diagnosing other inborn errors of metabolism, it is not the method of choice in the case of PKU due to the negative balance of potential benefits and drawbacks [7]. With the current effectiveness of disease detection protocols, scientific efforts are now focused not on improving the diagnostic process, but on improving the monitoring and treatment of patients with PKU. Advanced methods, such as a detailed assessment of the amino acid profile using HPLC and techniques utilizing “-omics” discoveries, offer new possibilities for creating individualized patient management plans [31].

Although we have data defining the micronutrient status in PKU and even studies on the plasma phospholipidome of PKU analyzed using hydrophilic interaction LC-MS/MS and gas chromatography–mass spectrometry, there is still a lack of reliable scientific works defining a detailed amino acid profile in patients with PKU [5,32,33,34]. So far, a detailed amino acid profile has been evaluated as one of the parameters when introducing new protein preparations or in people with late-diagnosed disease [35,36,37].

Especially important for PKU patients is tyrosine deficiency. This large neutral amino acid in PKU patients becomes an indispensable L-amino acid as a result of insufficient endogenous supply via PAH deficiency. Tyrosine is important not only in the production of thyroxin and melanin but also in the biosynthesis of neurotransmitters (L-dopa, dopamine, homovalinic acid). For this reason, tyrosine is supplemented in all L-amino acid formulas for PKU patients. Deficiency may indicate poorer compliance in the scope of formula intake and may result in problems with neurotransmission and, consequently, cognitive dysfunction. Lower concentrations of proline may also indicate problems with formula admission [7]. Indeed, reduced proline levels in PKU mothers have been associated with the insufficient consumption of Phe-free formula [38]. However, to be able to state the role of tyrosine and proline, further studies are needed that consider the actual intake of Phe-free formula and determine whether any patients have neurological problems (including neuroimaging techniques).

Moreover, the significantly lower kynurenine levels and kynurenine/tryptophan ratios in the studied group are also a matter of concern. The main precursor of kynurenine is tryptophan. Tryptophan, like tyrosine, is classified as one of the Large Neutral Amino Acids (LNAAs), which, apart from its obvious roles in the body’s protein homeostasis, reduces the toxic effects of Phe on the central nervous system through the ability to competitively block the transport of Phe through the blood–brain barrier [5,7,39]. Tryptophan plays a key role in the production of the neurotransmitter serotonin, melatonin, and, together with kynurenine, are precursors of niacin (vitamin B3). Kynurenine is associated with processes such as the body’s immune response, vasodilation in inflammatory processes, and oncogenesis. Decreased concentrations of kynurenine have been observed in patients with bipolar disorder [40,41]. Considering that in standard Phe-free mixtures, LNAAs constitute approx. 35–50% of the total number of L-amino acids, low concentrations of tryptophan and kynurenine may result either from the low consumption of the protein substitute by the tested patients or may indicate the need to increase LNAA’s supplementation in amino acid mixtures. However, before recommending an increase in tryptophan intake in Phe-free formulas, further studies are needed to investigate whether the intake of tryptophan is indeed low. Furthermore, it is important to note that LNAAs, including tryptophan, compete to cross the intestinal mucosa using the same L-type amino acid transporter 1. This competition can affect the absorption efficiency of each LNAA [42]. Thus, the low concentrations of tryptophan and kynurenine observed may also be a result of this competitive absorption process.

Another interesting finding is a significantly higher concentration of glycine in PKU patients. In addition to its role as a building block of proteins, glycine participates in glutathione synthesis and regulates gene expression and protein activity and configuration [43]. Zafra and Gimenez [44] highlight the important role of glycine in the process of neurotransmission. As an inhibitory neurotransmitter, particularly in the spinal cord and brainstem, glycine affects reflex reactions and the processing of sensory signals through glycine receptors [45]. Glycine is also a co-agonist of glutamate in the activation of N-methyl-D-aspartate receptors, thus participating in excitatory neurotransmission [46]. Generally, glycine is considered a non-essential amino acid biosynthesized in metabolic pathways, mainly from serine [47]. In our study, the levels of serine in the PKU group were similar to those obtained in the control subjects. The possible causes for this discrepancy could be related to higher protein intake and non-compliance.

Furthermore, in the present study, higher creatinine and asymmetric dimethylarginine levels in patients with PKU were revealed, which could be related to renal and cardiovascular risk. Charrière et al. [48], analyzing national health insurance claims data, found a more frequent prevalence of chronic renal failure and ischemic heart disease in patients with PKU. Similar findings related to renal and cardiovascular diseases were documented earlier based on North American and German data [13,15]. Hennermann et al. [49] found normal creatinine and cystatin C serum concentrations in all 67 studied PKU subjects. However, the glomerular filtration rate was diminished in 19% of them and inversely correlated with lifelong protein intake. Hermida-Ameijeiras et al. [50] documented that higher Phe levels were associated with an increased pulse wave velocity, which was not present in those subjects compliant with diet or under sapropterin treatment. Giret et al. [51] did not find any differences in plasma CRP levels and cytokine profiles between PKU patients and healthy controls. Kanzelmeyer et al. [52] documented similar L-arginine/asymmetric dimethylarginine plasma molar ratios in PKU patients and healthy controls. Additionally, Verduci et al. [53] suggested that the cardiovascular risk of compliant PKU children is not different from that of the healthy population. However, well-designed large studies are warranted to define PKU cardiovascular risk and its factors, including a balanced diet and metabolic compliance.

Our study aimed to characterize the intricate profile of metabolites using LC/MS techniques. We found significant alterations in the metabolome of PKU patients compared to healthy individuals. However, due to the small size of our study group, a limitation was the inability to interpret the results in light of adherence to dietary recommendations, assessed by average Phe levels and specific formula intake. Further research is necessary to gather more data and comprehensively interpret and apply findings to individualize dietary management in PKU patients.

In conclusion, the amino acid profiles of patients with PKU differ significantly from those of healthy peers, with potential clinical implications. These findings underscore the importance of metabolic testing in clinical practice and highlight the necessity for adequate dietary follow-up and adjustment. However, it is important to note that the sample size in this study is too small to draw definitive conclusions.

## Figures and Tables

**Table 1:** Characteristics of the studied groups.

		PKU Group		Control Group		*p* Value ^1^
		*N* (%)		*N* (%)		
Sex	female	11 (55.0)		31 (60.8)		0.789400
	male	9 (45.0)		20 (39.2)		
		**Median (Q1–Q3)**	**Mean ± SD (95%CI)**	**Median (Q1–Q3)**	**Mean ± SD (95%CI)**	***p* value ^2^**
Age (years)		24.5 (20.2–28.5)	24.6 ± 5.8 (22.0–27.1)	24.1 (21.7–28.0)	25.1 ± 4.5 (23.8–26.3)	0.715628
BMI (kg/m^2^)		21.3 (20.6–23.7)	22.0 ± 2.4 (21.0–23.1)	21.6 (19.8–23.4)	21.7 ± 3.1 (20.9–22.5)	0.649982
Annual median Phe concentration (mg/dL)		9.05 (5.75–13.72)	10.16 ± 5.70 (7.66–12.66)	-	-	-
Preceding year abnormal Phe results (%)		48.4 (6.23–100)	48.4 ± 41.9 (30.1–66.8)	-	-	-

BMI—body mass index; CI—confidence interval; PKU—phenylketonuria; Q—quartile; SD—standard deviation; ^1^ Fisher’s exact test; ^2^ The Mann–Whitney U test.

**Table 2:** Comparison of patients with phenylketonuria and the control group.

Analyte Name	PKU Group (*n* = 20)		Control Group (*n* = 51)		*p* Value ^1^
	Median (Q1–Q3)	Mean ± SD (95%CI)	Median (Q1–Q3)	Mean ± SD (95%CI)	
Acetylcarnitine	5.93 (4.99–8.06)	6.59 ± 2.57 (5.38–7.79)	5.60 (4.47–7.26)	6.06 ± 2.11 (5.47–6.65)	0.462324
Alanine	357 (300–440)	367 ± 94 (323–411)	339 (277–376)	338 ± 70 (319–358)	0.194422
Arginine	92.2 (73.4–108.5)	94.5 ± 28.9 (80.9–108.0)	84.4 (71.8–104.0)	86.1 ± 20.8 (80.2–91.9)	0.364063
Asparagine	43.4 (35.7–47.3)	40.7 ± 9.3 (36.4–45.1)	48.7 (44.0–52.7)	49.0 ± 7.1 (47.0–51.0)	0.000995
Aspartate	16.90 (13.85–20.05)	19.55 ± 8.94 (15.37–23.74)	19.10 (15.50–24.40)	20.38 ± 6.82 (18.46–22.30)	0.224560
Asymmetric dimethylarginine	0.517 (0.468–0.577)	0.521 ± 0.076 (0.485–0.556)	0.476 (0.410–0.495)	0.471 ± 0.079 (0.448–0.493)	0.009996
Carnitine	32.8 (28.2–40.10)	34.5 ± 8.6 (30.5–38.5)	30.0 (25.5–36.1)	31.3 ± 7.5 (29.2–33.4)	0.093998
Citrulline	31.1 (25.2–36.0)	30.9 ± 7.1 (27.6–34.3)	27.6 (24.2–32.7)	28.6 ± 6.2 (26.8–30.3)	0.139789
Creatinine	72.5 (65.5–83.2)	74.2 ± 13.7 (67.8–80.6)	62.9 (59.4–75.4)	68.1 ± 12.2 (64.7–71.6)	0.034920
Glutamate	50.5 (35.4–74.9)	66.00 ± 46.85 (44.08–87.93)	48.4 (37.2–67.8)	58.70 ± 31.00 (55.00–67.50)	0.974506
Glutamine	559 (491–653)	562 ± 128 (502–622)	582 (528–636)	582 ± 82 (559–606)	0.489986
Glycine	350 (329–451)	381 ± 110 (329–433)	263 (243–309)	282 ± 72 (262–302)	0.000012
Hexoses (including glucose)	4033 (3783–4493)	4061 ± 441 (3855–4268)	3883 (3487–4198)	3903 ± 507 (3760–4045)	0.169394
Histidine	84.2 (76.5–88.1)	83.9 ± 11.3 (78.7–89.2)	84.2 (76.3–93.4)	85.4 ± 12.2 (82.0–88.8)	0.691897
Isoleucine	69.7 (60.2–77.8)	68.9 ± 12.5 (63.1–74.8)	70.1 (62.0–83.3)	72.6 ± 16.8 (67.9–77.3)	0.522713
Kynurenine	1.200 (0.997–1.470)	1.309 ± 0.456 (1.096–1.522)	1.990 (1.690–2.390)	2.073 ± 0.502 (1.932–2.215)	0.000001
Leucine	134.5 (125.0–152.0)	137.4 ± 22.3 (127.0–147.9)	136.0 (115.0–160.0)	138.5 ± 36.4 (128.2–148.7)	0.832913
Lysine	158.0 (151.0–178.5)	165.2 ± 22.7 (154.6–175.9)	159.0 (139.0–186.0)	162.1 ± 32.6 (153.0–171.3)	0.547870
Methionine	21.6 (19.1–24.8)	21.8 ± 3.5 (20.2–23.4)	20.4 (18.8–23.9)	21.3 ± 4.25 (20.1–22.5)	0.398804
Ornithine	68.7 (68.7–50.1)	69.8 ± 23.8 (58.7–81.0)	74.1 (63.9–92.9)	81.3 ± 26.5 (73.9–88.7)	0.161583
Proline	143.5 (123.0–158.5)	149.6 ± 35.3 (133.1–166.2)	167.0 (142.0–200.0)	175.8 ± 51.8 (161.2–190.3)	0.011777
Putrescine	0.225 (0.165–0.295)	0.223 ± 0.086 (0.183–0.264)	0.161 (0.130–0.196)	0.166 ± 0.043 (0.154–0.178)	0.005532
Serine	123.0 (115.5–148.5)	129.3 ± 28.4 (116.0–142.7)	137.0 (118.0–158.0)	139.0 ± 25.1 (131.9–146.0)	0.214815
Serotonin	0.487 (0.277–0.588)	0.476 ± 0.276 (0.347–0.605)	0.557(0.364–0.729)	0.580 ± 0.273 (0.504–0.657)	0.159681
Threonine	109.0 (96.2–126.0)	117.0 ± 31.7 (102.1–131.8)	122.0 (101.0–139.0)	122.6 ± 27.6 (114.8–130.4)	0.205605
Tryptophan	62.4 (59.9–65.6)	63.0 ± 9.5 (58.6–67.5)	65.1 (58.0–70.6)	65.6 ± 10.3 (62.8–68.5)	0.435505
Tyrosine	44.8 (39.7–51.1)	47.4 ± 14.6 (40.5–54.2)	59.7 (50.2–65.8)	60.4 ± 14.5 (56.3–64.5)	0.000199
Valine	194 (180–220)	198.1 ± 28.9 (184.6–211.7)	202 (165–233)	201.0 ± 48.1 (187.5–214.6)	0.984700
Acetylcarnitine/Carnitine	0.175 (0.132–0.241)	0.198 ± 0.085 (0.159–0.238)	0.190 (0.144–0.244)	0.202 ± 0.081 (0.180–0.225)	0.739593
Asymmetric dimethylarginine/Arginine	0.006 (0.004–0.007)	0.006 ± 0.002 (0.005–0.007)	0.006 (0.004–0.006)	0.006 ± 0.002 (0.005–0.006)	0.348066
Branched-chain amino acids	399 (363–450)	405 ± 60 (377–433)	405 (353–463)	412 ± 98 (384–440)	0.974506
Citrulline/Ornithine	0.463 (0.375–0.585)	0.482 ± 0.159 (0.407–0.556)	0.358 (0.300–0.420)	0.374 ± 0.107 (0.343–0.404)	0.004274
Citrulline/Arginine	0.368 (0.274–0.439)	0.358 ± 0.126 (0.299–0.417)	0.352 (0.268–0.413)	0.348 ± 0.096 (0.321–0.375)	0.649968
Glucogenic amino acids	871 (805–974)	877 ± 182 (792–962)	730 (676–812)	759 ± 127 (724–795)	0.001776
Kynurenine/Tryptophan	0.021 (0.018–0.023)	0.021 ± 0.005 (0.018–0.023)	0.032 (0.027–0.036)	0.032 ± 0.006 (0.030–0.033)	<0.000001
Ornithine/Arginine	0.727 (0.546–0.934)	0.841 ± 0.485 (0.614–1.068)	0.879 (0.687–1.180)	1.017 ± 0.475 (0.883–1.150)	0.053576
Putrescine/Ornithine	0.003 (0.002–0.004)	0.003 ± 0.002 (0.003–0.004)	0.002 (0.002–0.003)	0.002 ± 0.001 (0.002–0.002)	0.003077
Serotonin/Tryptophan	0.007 (0.006–0.011)	0.008 ± 0.004 (0.006–0.010)	0.008 (0.006–0.011)	0.009 ± 0.005 (0.008–0.010)	0.403974

Q—quartile, CI—confidence interval, PKU—phenylketonuria, SD—standard deviation, ^1^ the Mann–Whitney U test.

**Table 3:** The AUC and *p* values along with the optimal cut-off values; sensitivity and specificity for the features statistically significantly discriminate between the studied groups; the optimal cut-off values were determined using the “closest to top-left corner” method.

Analyte Name	AUC Value	*p* Value	Optimal Cut-Off	Sensitivity	Specificity
Kynurenine/Tryptophan	0.919	<0.000001	0.026	0.90	0.84
Kynurenine	0.885	0.000001	1.505	0.80	0.90
Glycine	0.840	0.000012	320	0.85	0.80
Tyrosine	0.786	0.000199	47.8	0.70	0.84
Asparagine	0.756	0.000995	47.9	0.80	0.61
Glucogenic amino acids	0.749	0.001776	802	0.8	0.75
Citrulline/Ornithine	0.717	0.004274	0.419	0.65	0.75
Putrescine/Ornithine	0.715	0.003077	0.0025	0.65	0.67
Putrescine	0.724	0.005532	0.209	0.65	0.86
Asymmetric dimethylarginine	0.701	0.009996	0.5	0.65	0.78
Proline	0.689	0.011777	154	0.7	0.71
Creatinine	0.663	0.034920	64.2	0.8	0.53

AUC—area under the curve.

## Data Availability

The original contributions presented in the study are included in the article, further inquiries can be directed to the corresponding authors (J.M. & J.W.).
